# Current Updates on Simultaneous Prevention of COVID-19 and Monkeypox

**DOI:** 10.7759/cureus.99489

**Published:** 2025-12-17

**Authors:** Fariba Yazdanpanah

**Affiliations:** 1 General Preventive Medicine and Public Health, The University of Texas at Tyler, Tyler, USA

**Keywords:** cardiac complications, covid-19, immunization guidelines, mpox, myocarditis, pericarditis, prevention, update, vaccination

## Abstract

In 2022, the outbreak of monkeypox (Mpox) and the COVID-19 pandemic overlapped, raising concerns about the possibility that both viral infections might lead to the same heart problems, and questions about whether vaccination against both viruses could also increase cardiac complications caused by myocarditis or pericarditis. Because of these initial concerns, it was recommended that vaccines against both viruses be administered at separate intervals. However, recently, the Centers for Disease Control and Prevention (CDC) and the Advisory Committee on Immunization Practices (ACIP) have relaxed vaccination requirements while still emphasizing targeted precautions for high-risk individuals. This editorial outlines the updated national guidance issued by the CDC and ACIP and compares it with previous recommendations, providing clinicians with insight into how to safely and effectively implement these changes in clinical practice.

## Editorial

Introduction

In 2022, the world faced an outbreak of monkeypox (Mpox) as COVID-19 was still impacting the ability of healthcare systems around the world to respond effectively. While both viruses belong to separate families, each can lead to heart-related complications, including myocarditis and pericarditis, either through viral infection (directly) or vaccine-induced immune responses (indirectly). Reports from early clinical experiences showed that after the Mpox outbreak in 2022, there was insufficient awareness about the different types of heart-related complications, which caused concern among health professionals [1,2]. In my previous mini-review article on this topic, I examined the relationship between the two disease processes and provided precautionary recommendations from national guidelines to prevent further transmission during concurrent epidemics [3]. Since then, new guidance from the Centers for Disease Control and Prevention (CDC) and the Advisory Committee on Immunization Practices (ACIP) has revised vaccination timing recommendations as well as any advice on cardiac risk factors for vaccination and precautions related to Orthopoxvirus vaccines against Mpox and subsequent myocarditis and pericarditis [4,5]. In this medical editorial, the author aims to provide an updated perspective on Mpox immunization and to highlight the latest CDC and ACIP recommendations that now guide dual vaccination practices for Mpox and COVID-19.

Current guidance vs. previous recommendations

In my 2023 article, I noted ACIP’s cautious spacing between COVID-19 mRNA vaccines and Mpox immunization with Orthopoxvirus vaccines, either JYNNEOS^® ^or ACAM2000^®^, for four weeks, if Orthopoxvirus vaccines against Mpox have been given first [3]. Since then, the CDC and ACIP have updated their recommendations to include no minimum time interval between receiving COVID-19 vaccines and Orthopoxvirus vaccines against Mpox for anyone in the general population, except for young adult men and adolescents who need to receive both vaccines; a four-week interval between COVID-19 vaccines and Mpox immunization may be considered out of an abundance of caution [4]. The rationale behind this recommendation in young adult men and adolescents is due to the reported risk of pericarditis and myocarditis after receiving the ACAM2000^®^ and COVID-19 vaccines concomitantly, as well as the hypothesized risk of myocarditis and pericarditis following the JYNNEOS^®^ vaccine [4,5]. However, in critical circumstances with an increased risk of severe Mpox or COVID-19, regardless of age and gender, administration of Mpox and COVID-19 vaccines should not be delayed [4].

Additionally, CDC and ACIP recommend that the JYNNEOS^®^ vaccine be considered over ACAM2000^®^ if the COVID-19 vaccine and the Orthopoxvirus vaccine are to be administered concurrently, as the JYNNEOS^®^ vaccine has a more favorable safety profile and fewer contraindications [5]. Also, no minimum interval is required between receiving any COVID-19 vaccine and the JYNNEOS^®^ vaccine, regardless of which vaccine is administered first [4,5]. 

Clinical perspective

The updated immunization recommendations will affect how clinicians approach vaccinations in clinical practice, where COVID-19 and Mpox may be administered alongside. By eliminating the mandatory four-week interval between the two vaccines, vaccination logistics are less complicated and allow for the timely use of vaccinations at the high stage of transmission. Consideration continues for the risk of myocarditis in young adult men and adolescents, as this association was identified in early case reports and reaffirmed by the current national recommendations [1,4,5]. However, as evidence grows, vaccination intervals can be set more flexibly, provided that appropriate precautions for high-risk populations remain in place. From an editorial standpoint, several considerations are recommended: patient counseling regarding cardiac symptoms following vaccination continues to play a key role for individuals with known cardiac risk factors, along with considering the patient’s overall cardiac history when choosing the vaccine type (either JYNNEOS^®^ or ACAM2000^®^), given their different safety profiles. Lastly, ongoing evaluation of the integrated evidence related to Mpox and COVID-19 will be crucial as understanding of these diseases continues to evolve, allowing for improved prevention strategies and evidence-based clinical decision-making.

Conclusion

The concomitant appearance of COVID-19 and Mpox illustrates how multiple infectious diseases can present with similar yet distinct clinical features. As understanding of these diseases and their vaccines has grown, public health guidance has been revised to better support patient care. Current recommendations generally allow flexible timing between the two vaccines for these infectious diseases; however, certain groups are advised to take additional precautions. Staying informed about updated guidelines and monitoring post-vaccination symptoms in at-risk individuals can contribute to safe and effective vaccination strategies.
